# Molecular network characteristics and drug resistance analysis of 392 newly reported MSM HIV/AIDS cases in Chongqing, China

**DOI:** 10.3389/fpubh.2024.1308784

**Published:** 2024-06-06

**Authors:** Chongyang Bai, Tianyu Tan, Long Li, Rongrong Lu, Wei Zhang, Ling Ouyang, Guohui Wu, Chao Zhou

**Affiliations:** Chongqing Center for Disease Control and Prevention, Chongqing, China

**Keywords:** molecular network, transmitted drug-resistance, HIV-1, MSM, subtype

## Abstract

To comprehensively investigate the molecular transmission patterns of HIV-1 genotypes among men who have sex with men (MSM) in Chongqing, we employed 392 pol sequences of MSM to construct a phylogenetic tree and gene transmission network. Among the viral subtypes, CRF07_BC accounted for 73.2% (287/392) and CRF01_AE accounted for 20.7% (81/392), emerging as the predominant subtypes in this investigation. Additionally, we observed the presence of CRF55_01B, subtype B, CRF08_BC and other circulating recombinant forms. The HIV-1 molecular network was constructed with a gene distance threshold of 1.5%, resulting in an entry rate of 61.4% (241/392). Within the network, we identified a total of 23 molecular clusters, with the largest cluster being the CRF07_BC molecular cluster comprising 148 node values. Transmitted drug-resistance (TDR) mutations were found in 4.34% of the cases, with 1.79% associated with protease inhibitors (PIs), 0.51% with nucleoside reverse transcriptase inhibitors (NRTIs), and 2.55% with non-nucleoside reverse transcriptase inhibitors (NNRTIs). Statistical analysis indicated a higher enrollment rate in the HIV-1 molecular network among infected individuals with the CRF07_BC subtype, those identifying with same-sex sexual roles as “vers,” and individuals with higher education levels. This suggests the need for strengthened investigation and intervention in this population to prevent the formation of larger transmission clusters. Furthermore, continuous monitoring of the HIV-1 molecular dynamics network is necessary to promptly and accurately track changes in molecular epidemic characteristics.

## Introduction

The increasing burden of HIV/AIDS and comorbidities, along with the emergence of new HIV subtypes, circulating recombinant forms, drug mutations, and changing transmission networks, are likely to exert a significant impact on the HIV epidemic in China. Since the initiation of the National Free Antiretroviral Treatment Program (NFATP) by the Chinese government in 2003, the number of patients participating in this program has consistently increased, leading to broader antiviral medication coverage. However, this rise in treatment coverage has also been accompanied by an escalation in drug resistance. In China, according to the results of the fourth national HIV molecular epidemiology survey, the prevalence of transmitted drug resistance (TDR) was 3.80% in 2015 and increased to 4.40% in 2018 (1). Resistance mutation analysis revealed that among the newly reported HIV-infected individuals, the highest resistance rates were observed for non-nucleoside reverse transcriptase inhibitors, particularly nevirapine (NVP) and efavirenz (EFV). The specific resistance mutations identified were V179E/D/T, K103N, and V106I/M (2). The evolution of HIV drug resistance (HIVDR) poses a threat to the global expansion of antiretroviral treatment (ART) for HIV infection, thereby elevating the risk of ART failure. Consequently, conducting HIVDR surveys becomes crucial to determine the rate of TDR among ART-naive people living with HIV (PLWH). This survey provides essential baseline data for the successful implementation of ART programs, the mitigation of HIVDR incidence, and the formulation of effective public health policies aimed at curbing HIV prevalence.

The MSM community’s high mobility has significantly contributed to the widespread transmission of HIV across various locations and groups throughout China (3, 4). HIV viruses exhibit genetic variability, resulting in a growing number of recombinant subtypes. When coupled with the unique anal sexual behavior, frequent sexual activity, and multiple partners within the MSM population, this exacerbates the transmission of HIV (5–7). Chongqing is one of the areas most severely affected by HIV-1, with over 1,000 new cases of MSM infection reported each year, as documented by the China National Center for AIDS/STD Control and Prevention (NCAIDS). In recent years, Chongqing has exhibited a higher rate of sentinel and HIV Voluntary Counseling and Testing (VCT) infections compared to other Chinese provinces, such as Sichuan and Shaanxi (8, 9). Southwest China reported the highest pooled HIV-1 prevalence among MSM, with Chongqing city exhibiting the highest HIV prevalence (13.8, 95% CI: 12.8–14.9%) due to the region’s open attitudes toward homosexuality and sex (10, 11).

Because of the private nature of sexual behavior and the lengthy incubation period of AIDS, conducting an analysis of the social transmission network of HIV based solely on on-site epidemiological information faces numerous challenges and obstacles. To overcome these limitations, we have introduced HIV molecular transmission network analysis techniques, which enable the identification of associations between surveyed subjects at the molecular level. Previous studies have demonstrated that the sequence of the HIV pol coding region varies by less than 1% per year (12). Infected individuals who share transmission relationships exhibit greater genetic similarity in their viral gene sequences and tend to form clusters in phylogenetic analysis (13, 14).

This study enrolled a total of 392 newly reported MSM HIV-1-infected patients between 2019 and 2020. Phylogenetic analyses were conducted to examine the distribution and characteristics of HIV-1 subtypes, TDR, and molecular transmission clusters during this period. The objective of the study was to investigate the local transmission dynamics of prevalent HIV-1 strains within MSM populations and identify risk factors associated with network entry rates. The findings obtained from this study have the potential to inform targeted prevention and control strategies for the MSM population in Chongqing, China, thereby enabling more precise interventions.

## Methods

### Study participants

A cross-sectional investigation was conducted by the Chongqing Center for Disease Control and Prevention to study the MSM population. On-site surveys and follow-ups were carried out, and blood samples were collected from newly reported MSM individuals with HIV/AIDS between 2019 and 2020. The inclusion criteria were as follows: individuals who reported their cases between January 1, 2019, and December 31, 2020; males aged 18 years or older; individuals who had not initiated antiviral therapy at the time of the survey; individuals who were willing to cooperate and did not have any other serious concurrent illnesses; individuals who understood the purpose of the survey and provided informed consent.

In China, AIDS is a national legal infectious disease, and all newly diagnosed cases of AIDS need to be reported in “China AIDS Comprehensive Prevention and Control Data Information System.” And all participants will be screened and matched using personal information in this system to ensure that all subjects included in the study are newly reported HIV cases. A total of 448 participants met the requirements for the survey.

### Sample collection and RNA extraction

For eligible study participants, venous blood samples of 5 mL were collected in anticoagulation tubes containing EDTA-K by trained professionals. The blood samples were then processed to separate the plasma, which was subsequently stored in a low-temperature freezer at −80°C. To extract HIV-1 RNA from the plasma samples of infected patients, a Thermo Scientific KingFisher Flex high-throughput automated nucleic acid extractor was employed.

### Amplification and sequencing

Nucleic acid amplification was performed using Takara PrimeScript One Step RT-PCR and Tengen 2 × Taq PCR (premixed) reagents. The protease (PR) gene region and the first 300 amino acids of the reverse transcriptase (RT) gene region were amplified using the pol gene region of the international standard strain HXB2 (2,253–3,553 nt) as a reference. A nested polymerase chain reaction (PCR) technique was employed to amplify characteristic fragments of the pol region of the HIV-1 virus with commercial primers, following previously published methods (15). The PCR products were then sent to Beijing Nordson Genome Research Center Co., Ltd. for purification and subsequent gene sequencing. The amplification primers and sequencing primers were provided by Beijing Nordson Genome Research Center Co.

### Sequence splicing and subtype identification

The resulting pol sequence was spliced using the Sequencher 5.4.6 analytical program. Subsequently, the sequence underwent editing and correction using the BioEdit Sequence Alignment Editor. All sequences used in this study have been uploaded the sequence to the National Microbiological Data Center of China (No:NMDC10018715), which can be accessed through the link.^1^ Genotype determination was performed using MEGA X software. To align all assembled sequences with reference sequences from the HIV sequence database of Los Alamos National Laboratory in the United States, the Clustal W algorithm in MEGA X was employed. Manual verification of the alignment was conducted in Bioedit (16).

FastTree software was utilized to estimate an approximate maximum likelihood phylogenetic tree for the pol sequences. The GTR + G + I nucleotide substitution model was applied (17), and the Shimodaira-Hasegawa (SH) test within the software was used to calculate the node (branch point) value of the evolutionary tree. Clades with SH-like support ≥0.90 were defined (18, 19). The preliminary determination of genotype was achieved by clustering the sample sequences with international reference strains. The results were further validated using the online analysis tool HIV Databases BLAST.^2^ Recombinant identification was performed using the Recombinant Identification Program (RIP) (20) implemented in the HIV sequence Database.^3^ The phylogenetic tree was visualized using FigTree v1.4.3^4^ (20). If the genotype of a sequence could not be determined through phylogenetic tree analysis and HIV Databases, it was classified as a Unique Recombinant Form (URF).

### Drug resistance mutation analysis

The distribution of HIV drug resistance mutations (DRMs) among the study subjects was analyzed based on 25 commonly used antiretroviral therapeutic drugs listed in the HIVDB database^5^ of Stanford University, USA. The analysis aimed to assess drug resistance related to nucleoside reverse transcriptase inhibitors (NRTIs), non-nucleoside reverse transcriptase inhibitors (NNRTIs), and protease inhibitors (PIs) (21, 22).

### Molecular network analysis

The hyphy 2.2.4 software was employed to estimate the genetic distances between clusters in the TN93 model. Molecular transmission clusters were generated using Cytoscape 3.9.0 (23). A threshold of 0.5% signifies a nucleotide sequence of 1,000 bp, representing 5 different nucleotides. This threshold suggests the presence of a newly infected molecular cluster, as the maximum time for the virus strains to evolve to 5 different nucleotides is approximately 2–3 years. Conversely, a threshold of 1.5% represents a nucleotide sequence of 1,000 bp, corresponding to 15 different nucleotides. In this case, the threshold indicates long-term infected molecular clusters, as the maximum time for the virus strains to evolve to 15 different nucleotides is around 7–8 years (24).

### Statistical analysis

The professional investigators at the STD/AIDS counseling clinic were responsible for recording the questionnaire, which collected basic demographic and epidemiological information from the survey respondents. The epidemiological information encompassed marital status, age, occupation, culture, and sexual roles (“Top”—The individual who assumes the penetrative role during sexual activity. “Bottom”—The individual who assumes the receptive role during sexual activity. “Vers”—Individuals who are flexible and may assume either the top or bottom role, depending on the context or preference) in male-to-male sex. Statistical analysis was conducted using SPSS 26 software (IBM, Chicago, IL, United States). Crude odds ratio (COR) with 95% confidence interval (CI) and adjusted odds ratio (AOR) with 95% CI were determined using univariate and multivariate logistic regression models separately in order to analyze the factors influencing entry into the molecular transmission network. A significance level of *p* < 0.05 was used to determine statistical significance.

## Results

### Demographic characteristics of the study participants

A total of 392 pol region sequences were successfully obtained from 445 samples after nucleic acid amplification and gene sequencing (88.09% sequencing success rate). The average age of those individuals was 32.2 years (ranging from 18 to 69 years). Of these subjects, 79.8% (313/392) were unmarried, 12.8% (50/392) were married, and 7.4% (29/392) were divorced or widowed. From the education aspect, college and above accounted for 59.70% (234/392). The sex role survey showed that 42.3% (166/392) were the “Top,” 23.3% (91/392) were the “Bottom,” and 34.2% (135/392) were the “Verse.” The majority of the subjects (72.5%, 284/392) had CD4+ T lymphocyte count ≤350 cells/μL before treatment (Table 1).

**Table 1:** Characteristics of 392 newly reported cases of MSM HIV/AIDS in Chongqing, China.

Variables	Number	Percent
Total	392	100.00
Age (years)		
18–30	215	54.80
31–40	98	25.00
>40	79	20.20
Occupation		
workers and farmers	36	9.20
Unemployment	73	18.60
Business services	255	65.10
Students	28	7.10
Education level		
Primary and junior high school	58	14.80
High school	100	25.50
College degree and above	234	59.70
CD4+ T lymphocyte count before treatment (cells/μL)		
<200	150	38.30
200–350	134	34.20
≥350	71	18.10
Miss^#^	37	9.40
Marital status		
Unmarried	313	79.80
Married	50	12.80
Divorced/widowed	29	7.40
Sex Roles		
“Top”	166	42.30
“Bottom”	91	23.30
“Vers”	135	34.40
Virus subtypes		
CRF07_BC	287	73.20
CRF01_AE	81	20.70
CRF55_01B	12	3.06
CRF08_BC	5	1.26
B	4	1.02
URF	3	0.76

^#^CD4+ T lymphocyte testing not conducted or results not available.

### The prevalence of TDR

Among the 392 individuals included in the sequencing analysis, 17 individuals (4.34%) were found to be infected with HIV strains that harbored at least one drug resistance mutation (DRM). The prevalence of TDR to NNRTIs was 2.55%, followed by TDR to NRTIs at 0.51%, and TDR to PIs at 1.79%. The prevalence of dual-class TDR was 0.06% for both PI and NNRTI, as well as for NRTI and NNRTI. The most common DRMs associated with NNRTIs, NRTIs, and PIs were K103N/ E138A, L74LI/M184I and Q58E, respectively (Table 2).

**Table 2:** Distribution of DRMs in 392 newly reported cases of MSM HIV/AIDS in Chongqing, China.

DRMs	Number	TDR%	Drug^a^
Total	17	4.34	
NNRTIs			
K103N	3	0.77	EFV/NVP
Y181YC	1	0.26	EFV/ETR/NVP/RPV
E138A	3	0.77	RPV
E138EG, V179E	1	0.26	EFV/ETR/NVP/RPV
V106VI, V179D	1	0.26	EFV/ETR/NVP/RPV
V179IL	1	0.26	RPV
NRTIs			
L74LI	1	0.26	ABC/DDI
M184I	1	0.26	ABC/FTC/3TC
PIs			
G73GRS	1	0.26	IDV/r, SQV/r, NFV
M46MI	1	0.26	NFV
Q58E	3	0.77	TPV/r
M46L	2	0.51	NFV

^a^Drug indicates the 25 drug available on the HIVDB database at Stanford University. EFV, efavirenz; NVP, nevirapine; DDI, desoxynivalenol; D4T, stavudine; FTC, emtricitabine; DRV, darunavir; 3TC, lamivudine; ABC, abacavir; NFV, nelfinavir; IDV, indinavir; SQV/r, saquinavir + ritonavir; TPV/r, tipranavir + ritonavir.

### Identification and characterization of HIV-1 subtype distribution and molecular network

**Figure 1:**
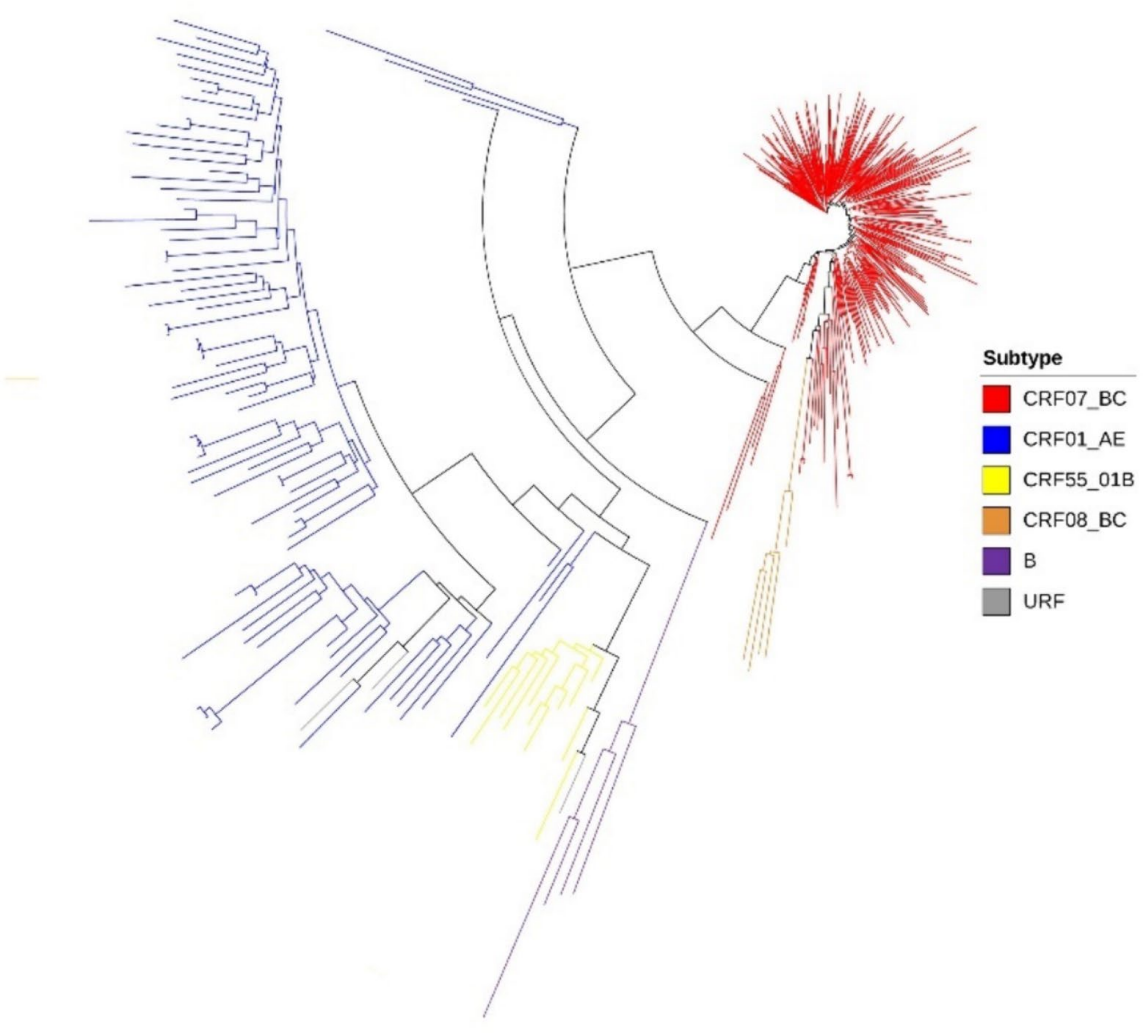
Phylogenetic tree of lineages, the phylogenetic tree was constructed using approximately-maximum-likelihood (ML) method based on *pol* region (HXB2: 2, 253 to 3, 306 nt) in Fast Tree 1.4.3. The nucleotide substitution mode was GTR + G + I.

Among the study subjects, a total of 392 samples were successfully sequenced and genotyped, accounting for 88.09% (392/445) of the total sample size. Analysis using the approximately maximum-likelihood (ML) tree in Fast Tree 2.3 revealed that the dominant subtype was CRF07_BC, accounting for 73.20% (287/392) of the samples. This was followed by CRF01_AE at 20.70% (81/392), CRF55_01B at 3.06% (12/392), and subtype CRF08_BC at 1.26% (5/392) (Table 1). Additionally, other subtypes including B, and URFs were also detected in sequential order. In the phylogenetic tree, the differing lengths of tree scales among the various viruses resulted in separate clustering of viral sequences with different genotypes (Figure 1).

**Figure 2:**
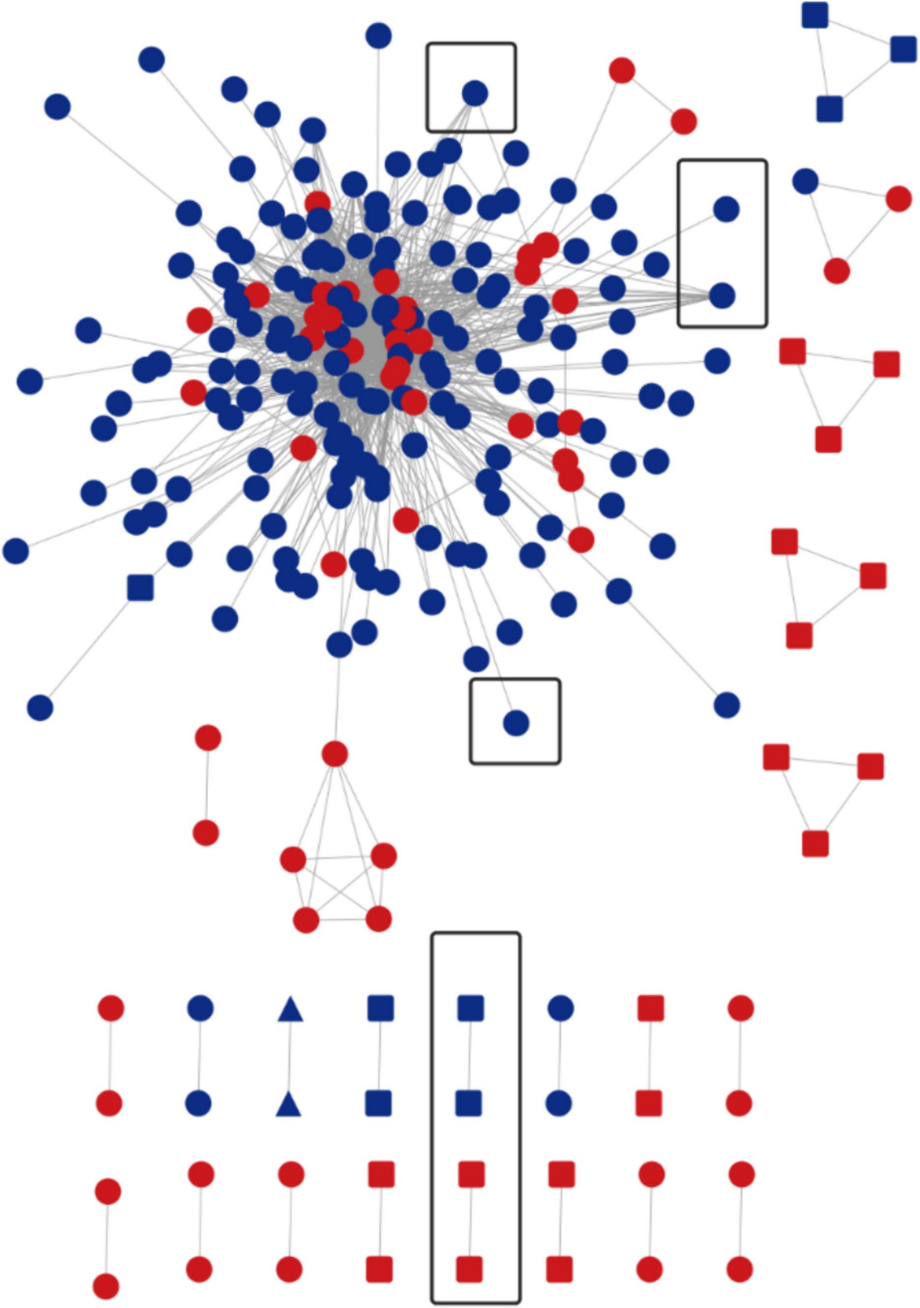
Molecular network at a gene distance threshold of 1.5%. Nodes and molecular clusters representing sequences at a gene distance threshold of 0.5% were highlighted in red. The presence of a circle indicated the genotype of HIV at that particular node was CRF07_BC. A rounded rectangle represented the genotype CRF01_AE, while a triangle denoted CRF55_01B. Additionally, black boxes were used to indicate transmitted drug-resistance clusters or nodes.

The HIV-1 molecular network was constructed according to the gene distance threshold of 1.5%. A total of 241 nodes entered the network, with an entry rate of 61.4%. And a total of 23 molecular clusters (≥2 nodes, each node representing a sequence) were found. Visualization by Cytoscape 3.9.0 software revealed several large MSM transmission clusters in the molecular network, with the largest MSM molecular cluster containing 148 nodes, where the viral genotype represented by each node was CRF07_BC. Next, a gene distance threshold of 0.5% was chosen to construct the molecular network, and a total of 72 nodes entered the network, with an entry rate of 18.4%. A total of 27 molecular clusters were formed, with the largest one containing 12 nodes. We can also find that 39 nodes in the largest MSM molecular cluster are new additions, and the number of nodes directly or indirectly associated with the propagation network of long-term infections reaches 41, accounting for 56.94% (41/72). By drug resistance transmission cluster analysis, a total of 8 transmission resistant nodes were found in this network, with the main virus subtypes CRF07_BC and CRF01_AE, of which 6 nodes were in the long-term infection transmission network and 2 nodes were in the newly infected network (Figure 2).

### Analysis of risk factors of transmitted molecular network

In the multivariable logistic regression model, education level (high School, AOR = 3.535, 95% CI 1.597–7.828; college degree and above, AOR = 2.767, 95% CI 1.389–5.512.), sex role (“Vers,” AOR = 2.409, 95% CI 1.366–4.246.), and virus subtypes (CRF01_AE, AOR = 0.106, 95% CI 0.059–0.192; CRF55_01B, AOR = 0.051, 95%CI 0.010–0.248.) were significantly associated with being in clusters compared to those who were not in clusters (Table 3).

**Table 3:** Analysis of factors influencing transmission within clusters of new reported 392 cases of HIV/AIDS in MSM in Chongqing, China.

Variables	Total (*n* = 392)	In cluster (n = 241; 61.4%)	COR (95%CI)	*p*-value	AOR (95%CI)	*P*-value
Age						
18–30	215	135 (62.8%)	1.000	0.066		
31–40	98	66 (67.3%)	1.222 (0.738–2.025)	0.436		
>40	79	40 (50.6%)	0.608 (0.361–1.023)	0.061		
Occupation						
workers and farmers	36	16 (44.4%)	1.000		1.000	
Unemployment	73	44 (60.3%)	1.897 (0.846–4.252)	0.120	1.132 (0.440–2.915)	0.797
Business Services	255	163 (63.9%)	2.215 (1.094–4.484)	0.027	1.765 (0.754–4.129)	0.190
Students	28	18 (64.3%)	2.250 (0.816–6.207)	0.117	2.651 (0.746–9.425)	0.132
Education level						
Primary and junior high school	58	25 (43.1%)	1.000		1.000	
High school	100	64 (64.0%)	2.347 (1.212–4.544)	0.011	3.535 (1.597–7.828)	0.002
College degree and above	234	152 (65.0%)	2.447 (1.363–4.392)	0.003	2.767 (1.389–5.512)	0.004
CD4^+^ T cells (cells/μL)						
<200	150	94 (62.7%)	1.000			
200–350	134	84 (62.7%)	1.001 (0.618–1.620)	0.997		
>350	71	35 (49.3%)	0.579 (0.327–1.025)	0.061		
Miss	37	28 (75.7%)	1.853 (0.816–4.211)	0.141		
Marital status						
Unmarried	313	197 (62.9%)	1.000			
Married	50	26 (52.0%)	0.638 (0.350–1.163)	0.142		
Divorced/widowed	29	18 (62.1%)	0.964 (0.440–2.111)	0.926		
Sex roles						
“Top”	166	93 (56.0%)	1.000		1.000	
“Bottom”	91	55 (60.4%)	1.199 (0.713–2.017)	0.494	1.669 (0.881–3.160)	0.116
“Vers”	135	93 (68.9%)	1.738 (1.080–2.798)	0.023	2.409 (1.366–4.246)	0.002
Virus subtypes						
CRF07_BC	287	215 (74.9%)	1.000		1.000	
CRF01_AE	81	24 (29.6%)	0.14 (0.082–0.244)	<0.001	0.106 (0.059–0.192)	<0.001
CRF55_01B	12	2 (16.7%)	0.06 (0.014–0.313)	0.001	0.051 (0.010–0.248)	<0.001
Others	12	0 (0.0%)	–	0.998	–	0.998

## Discussion

In this study, a cross-sectional survey was conducted to assess the molecular epidemiological status of HIV-1. The focus was on tracking the characteristics and distribution of HIV-1 genotypes, TDR, DRMs, and molecular transmission clusters among newly diagnosed HIV-infected patients in Chongqing. Additionally, the study aimed to explore the factors influencing the rate of entry into the molecular network.

In China, the overall prevalence of TDR in the ART-naïve population was reported to be 3.8% (30), which is slightly lower than the TDR prevalence of 4.34% observed in our study. The distribution of DRMs in our study was found to be similar to that of Yunnan, Heilongjiang, and Sichuan provinces in China (9, 25, 26). Specifically, the prevalence of TDR to NNRTIs (2.55%) was higher compared to NRTIs (0.51%) and PIs (1.79%). This observation can be attributed to the low genetic barrier to resistance of NNRTIs, where a single major mutation often leads to multiple and high-level resistance to NNRTI drugs (27–29). The common DRMs for NNRTIs in our study were K103N (30%) and E138A (30%), which aligns with previous analyses of HIV-1 transmission resistance in newly diagnosed HIV-infected patients in Sichuan Province (30). This finding is related to the widespread use of NNRTI-based (EFV/NVP) first-line treatment in China for over a decade, consistent with data from low- and middle-income countries (31–33). Moving forward, it is essential to strengthen drug resistance monitoring in the MSM population during antiviral treatment. Close attention should be paid to the spread of drug-resistant strains, with concerted efforts to reduce the further proliferation of drug-resistant variants.

Molecular network analysis techniques have become increasingly important in AIDS research in various countries (34–36). They provide valuable insights into the transmission dynamics of HIV-1, aiding in the identification of key transmission clusters, and drug resistance transmission clusters and associated risk factors (37, 38). By comparing the newly infected molecular network with the long-standing infected molecular network, certain key transmission clusters and network segments are identified, which have experienced rapid expansion within a short timeframe. Given the low prevalence of TDR within the MSM population, larger transmission drug-resistant clusters were not observed. However, the transmission drug-resistant clusters mainly consisted of cases with long-term infections. This highlights the importance of enhancing surveillance for drug-resistant transmission clusters and DRMs within the molecular network.

The analysis of subtypes revealed that CRF07_BC and CRF01_AE were the predominant subtypes among the MSM population in Chongqing. This finding is consistent with previous studies conducted in Anhui and Guangzhou (39, 40), and is also in line with the national molecular epidemiological subtype distribution in 2015 (41). CRF07_BC formed 8 molecular clusters, with the largest cluster containing 148 nodes, indicating significant aggregation within the MSM population. Figure 2 demonstrates that the molecular cluster formed at a 0.50% threshold is associated with the large cluster of MSM CRF07_BC formed at a 1.50% threshold. This suggests that the CRF07_BC molecular cluster has been gradually expanding over time. To achieve the goal of reducing new infections, it is recommended to implement timely and focused interventions, conduct testing, and promote condom use within this molecular cluster. By identifying the key transmitters in this cluster, targeted strategies can be developed to effectively control and prevent the further spread of CRF07_BC and reduce the incidence of new infections.

In addition to CRF07_BC, it is important to highlight the significance of CRF01_AE and CRF55_01B. Initially, the transmission of CRF01_AE was primarily observed in the eastern coastal regions and southwestern border provinces of China, predominantly affecting heterosexual populations. However, recent findings from the Chinese Center for Disease Control and Prevention indicate that CRF01_AE has rapidly become the most prevalent HIV-1 viral subtype, spreading widely across geographic regions and high-risk populations, particularly among Chinese MSM (42–44).

Similarly, CRF55_01B initially emerged and spread rapidly within the MSM population in Guangdong, China. It has now disseminated to multiple provinces throughout the country (40, 45). The rapid expansion of CRF55_01B highlights the need for ongoing monitoring of this molecular cluster and the implementation of effective interventions to control its transmission within the population.

The results of statistical analysis showed that the variables were CRF07_BC subtype and sex role as “vers” were statistically significant. Further analysis of the “vers” population revealed that this population was generally young and unmarried, and that the viral subtype CRF07_BC was present in both the new-onset and long-term infection networks. A total of 7 individuals with DRMs in the “vers” population, including 4 individuals in the molecular network, suggest that we should strengthen the monitoring of genotypic drug resistance in the “vers” population in the molecular network. The multi-factor analysis of education level shows that the enrollment rate of both high school and above education is high, the possible reason for this result may be because the source of this survey is concentrated in the young group in the main city of Chongqing, and the education level of the young group is generally high. The study showed that new MSM infections in this trial were characterized by young age, high proportion of unmarried people, and good education level. This is similar to the distribution of demographic information in the previous MSM HIV/AIDS transmission cluster analysis in Shanghai and Chongqing (42, 46). The risk factors for entry into the network suggest that we need to strengthen MSM investigation and intervention. Identifying additional risk factors and intervening in a timely manner can stop further expansion of the transmission cluster.

Presently, one of the formidable challenges in the HIV epidemic in China is the substantial proportion of diagnoses made at advanced stages of infection. As highlighted by previous research (47), the incidence of late-stage HIV diagnoses in Chongqing persistently exceeds the national average, underscoring a critical public health issue that necessitates immediate intervention. In the context of the present study, a significant majority of the participants (72.5%, 284/392) exhibited a CD4+ T lymphocyte count of ≤350 cells/μL prior to initiating treatment. Given the complexities associated with accurately determining the timing of infection, this variable was excluded from the current analysis. Looking forward, our research will integrate HIV new infection testing methodologies and epidemiological assessments to more precisely estimate the time of infection, thereby enhancing our understanding of the relationship between CD4+ T lymphocyte count and the timing of HIV acquisition.

## Conclusion

We show the distribution and features of HIV-1 subtypes, TDR, DRMs, and molecular transmission clusters in the MSM population of Chongqing, China, and investigate the risk factors that influence cluster formation. Although the overall incidence of TDRs in this study was low, it is nevertheless vital to underline the need of genotypic and molecular drug resistance surveillance, with a focus on critical drug resistance mutations. Following that, we will aim to support the creation and implementation of an HIV-1 molecular dynamic surveillance network in Chongqing.

## Data availability statement

The datasets presented in this study can be found in online repositories. The names of the repository/repositories and accession number(s) can be found at: https://nmdc.cn/resource/genomics/project/detail/NMDC10018715.

## Ethics statement

The studies involving humans were approved by the institutional review board of Chongqing Center for disease control and prevention. The studies were conducted in accordance with the local legislation and institutional requirements. The participants provided their written informed consent to participate in this study.

## Author contributions

CB: Writing – original draft, Writing – review & editing. TT: Writing – original draft, Writing – review & editing. LL: Writing – original draft, Writing – review & editing. RL: Writing – original draft, Writing – review & editing. WZ: Writing – original draft, Writing – review & editing. LO: Writing – original draft, Writing – review & editing. GW: Writing – original draft, Writing – review & editing. CZ: Writing – original draft, Writing – review & editing.

## Footnotes

^1^ https://nmdc.cn/resource/genomics/project/detail/NMDC10018715

^2^ https://www.hiv.lanl.gov/content/sequence/BASICBLAST/basicblast.html

^3^ http://www.hiv.lanl.gov/content/sequence/RIP/RIP.html

^4^ http://tree.bio.ed.ac.uk/software/figtree/

^5^ https://hivdb.stanford.edu/
