# Supplementary material for: Molecular network characteristics and drug resistance analysis of 392 newly reported MSM HIV/AIDS cases in Chongqing, China
Source: Front Public Health. 2024 Jun 6;12:1308784. doi: 10.3389/fpubh.2024.1308784 (PMC11187242; doi:10.3389/fpubh.2024.1308784)
Supplement: Supplementary file 1 [file Data_Sheet_1.docx]

1. the PCR/primer details

The HIV-1 RNA pol region encodes regions of the reverse transcriptase and protease genes (HXB2: 2253–3553nt), which were amplified using nested PCR technique with commercial primers, following previously published methods^15^. To provide further clarification, we have included this supplementary explanation in the manuscript (line 106). The specific primers and amplification parameters were as follows:

First-round PCR:

Outer upstream primer PRTM-F1:

F1a-5’-TGAARGAITGYACTGARAGRCAGGCTAAT-3’; HXB2 2057-2085

F1b-5’-ACTGARAGRCAGGCTAATTTTTTAG-3’; HXB2 2068-2092

Outer downstream primer RT-R1: 5’-ATCCCTGCATAAATCTGACTTGC-3’; HXB2 3370-3348

Second-round PCR:

Inner upstream primer PRT-F2: 5’-CTTTARCTTCCCTCARATCACTCT-3’; HXB2 2243-2266

Inner downstream primer RT-R2: 5’-CTTCTGTATGTCATTGACAGTCC-3’; HXB2 3326-3304

Nested PCR amplification technique was employed. In the first round of PCR, primers were used in a 25 μl amplification system with One Step Reverse Transcription PCR reagents (Takara, Dalian, China) to amplify the HIV pol gene region. The specific reaction conditions were as follows: 50°C for 45 minutes; 94°C for 2 minutes; 94°C for 15 seconds, 55°C for 20 seconds, 72°C for 2 minutes, 35 cycles; 72°C for 10 minutes; incubation at 4°C. Subsequently, in the second round of PCR, primers were used in a 50 μl amplification system with 2× Taq PCR MasterMix (Tiangen, Beijing, China) to amplify the target gene fragment. The specific reaction conditions were as follows: 94°C for 4 minutes; 94°C for 15 seconds, 55°C for 20 seconds, 72°C for 2 minutes, 35 cycles; 72°C for 10 minutes; incubation at 4°C.

Using the Sanger sequencing method, appropriate sequencing primers were selected with the following specific parameters:

Forward sequencing primers:

PRT-F2: 5’-CTTTARCTTCCCTCARATCACTCT-3’ (2243-2266)

SeqF3: 5’-AGTCCTATTGARACTGTRCCAG-3’ (2556-2577)

SeqF4: 5’-CAGTACTGGATGTGGGRGAYG-3’ (2869-2889)

Reverse sequencing primers:

RT-R2: 5’-CTTCTGTATGTCATTGACAGTCC-3’ (3326-3304)

SeqR3: 5’-TTTYTCTTCTGTCAATGGCCA-3’ (2639-2619)

SeqR4: 5’-TACTAGGTATGGTAAATGCAGT-3’ (2952-2931).

15 Liao, Lingjie et al. “The prevalence of transmitted antiretroviral drug resistance in treatment-naive HIV-infected individuals in China.” Journal of acquired immune deficiency syndromes (1999) vol. 53 Suppl 1,Suppl 1 (2010): S10-4. doi:10.1097/QAI.0b013e3181c7d363

Regarding the gel electrophoresis images, we can provide a selection of them:


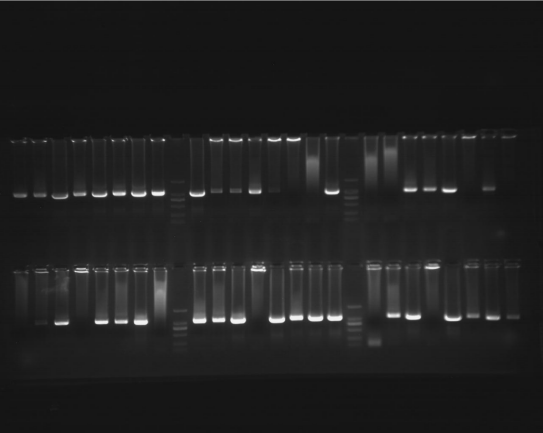


2. Stratified analysis among variables of virus subtypes, education level, and vers

In our research, we conducted a stratified analysis of variables such as CRF07BC, education level (high school and college degree), and vers. The chi-square test results indicate that after stratification, the P-values for all variables are <0.05, as detailed in the table below. This suggests a relatively independent relationship among these variables with no apparent confounding effects.

| **Variables** | CRFO7_BC | **χ^2^** | **P-value** |
| --- | --- | --- | --- |
| **Education level** |  | 101.693 | ＜0.001 |
| Primary and Junior High School | 44 |  |  |
| High School | 68 |  |  |
| College degree and above | 175 |  |  |
| **Sex Roles** |  | 20.265 | ＜0.001 |
| “Top” | 125 |  |  |
| “Bottom” | 63 |  |  |
| “Vers” | 99 |  |  |

.

| **Variables** | “Vers” | **χ^2^** | **P-value** |
| --- | --- | --- | --- |
| **Education level** |  | 38.800 | ＜0.001 |
| Primary and Junior High School | 21 |  |  |
| High School | 36 |  |  |
| College degree and above | 78 |  |  |
| **Virus subtypes** |  | 179.578 | ＜0.001 |
| CRFO7_BC | 99 |  |  |
| CRF01_AE | 28 |  |  |
| CRF55_01B | 4 |  |  |
| Others | 4 |  |  |

| **Variables** | high school and college degree | **χ^2^** | **P-value** |
| --- | --- | --- | --- |
| **Virus subtypes** |  | 437.473 | ＜0.001 |
| CRFO7_BC | 243 |  |  |
| CRF01_AE | 72 |  |  |
| CRF55_01B | 11 |  |  |
| Others | 8 |  |  |
| **Sex Roles** |  | 26.036 | ＜0.001 |
| “Top” | 148 |  |  |
| “Bottom” | 72 |  |  |
| “Vers” | 114 |  |  |

3. Associations between CD4 count, HIV-1 subtypes and DRM

The chi-square test results indicate that these variables have no associations with each other (P>0.05).

| **Variables** | **CD4<200** | **200~350** | **≥350** | **χ^2^** | **P-value** |
| --- | --- | --- | --- | --- | --- |
| **Virus subtypes** |  |  |  | 12.097 | P=0.060 |
| CRFO7_BC | 108 | 101 | 49 |  |  |
| CRF01_AE | 36 | 19 | 18 |  |  |
| CRF55_01B | 4 | 5 | 3 |  |  |
| Others | 2 | 9 | 1 |  |  |

| **Variables** | **DRMS** | **χ^2^** | **P-value** |
| --- | --- | --- | --- |
| **Virus subtypes** |  | 6.203 | 0.102 |
| CRFO7_BC | 8 |  |  |
| CRF01_AE | 7 |  |  |
| CRF55_01B | 1 |  |  |
| Others | 1 |  |  |
